# Endovascular Management of a Giant Renal Artery Aneurysm With Arteriovenous Malformation: A Case Report

**DOI:** 10.7759/cureus.88489

**Published:** 2025-07-22

**Authors:** Jochen Gerstner Saucedo, Isabel Carmona, Davood Abdollahian, Orlando Diaz

**Affiliations:** 1 Diagnostic Radiology, University of Colorado Anschutz Medical Campus, Aurora, USA; 2 Medicine, Universidad ICESI, Cali, COL; 3 Interventional Radiology, Houston Methodist Hospital, Houston, USA; 4 Interventional Neuroradiology, Houston Methodist Neurological Institute, Houston, USA

**Keywords:** arteriovenous malformation, embolization, endovascular repair, renal artery aneurysm, vascular intervention, vascular malformation, visceral aneurysm

## Abstract

Renal arteriovenous malformations (rAVMs) are rare vascular abnormalities where there is a direct connection between the renal artery and vein, which circumvents the normal capillary network. The endovascular approach is preferred due to its minimally invasive nature, shorter hospital stay, and lower risk of complications in comparison to open surgery. Open surgery is therefore indicated in emergent situations such as arteriovenous malformation (AVM) rupture, hemodynamic instability, failure of endovascular therapy, or complex vascular anatomy that makes the endovascular approach unfeasible. However, endovascular management can be complicated by anatomic variants, such as renal artery duplication, and concomitant pathology, like fibromuscular dysplasia (FMD). This case demonstrates how a multimodal embolization approach, including coil deployment and flow-directed liquid embolic under balloon occlusion, can achieve technical success and favorable clinical outcomes with minimal risk. A 53-year-old woman with a history of hypertension presented with chronic abdominal pain. Physical examination revealed a right-sided abdominal bruit. A renal ultrasound revealed a 3.8 cm saccular aneurysm with internal low-resistance arterial flow near the renal hilum, which was initially interpreted as a renal artery aneurysm. In order to better delineate anatomy, an abdominal computed tomography angiogram was obtained, which showed a direct connection between a single segmental branch and a large, saccular venous aneurysm, compatible with a Yakes type I rAVM. It also showed several additional abnormalities, including a duplicated right renal artery with a “beads on a string” morphology suggestive of concomitant FMD and a small arterial aneurysm. Because of the size of her venous aneurysm and concern for hemorrhage, she was referred for surgical evaluation. Surgical excision/ligation was deemed too invasive, so the patient was then referred to interventional radiology for endovascular treatment with coil embolization of the venous sac and balloon-assisted Onyx embolization of the nidus. The technique involved staged coil embolization of the venous sac followed by Onyx injection into the nidus under balloon occlusion to achieve flow control and precise embolic delivery. This case highlights recent technical advancements, such as dual lumen balloon microcatheters to control inflow and the liquid embolic Onyx, which facilitated successful endovascular management of a rAVM with associated venous aneurysm. While coils remain the most commonly used embolic material for rAVMs, they primarily occlude the feeding vessels without directly addressing the nidus, which can result in incomplete treatment and potential recurrence. That is why, by pairing them with Onyx, we can treat the feeding vessels and effectively penetrate and fill the nidus, offering a more comprehensive and durable occlusion.

## Introduction

Arteriovenous malformations (AVMs) are abnormal connections between arteries and veins that bypass the capillary system, often through a network of intervening vessels, known as the nidus. The nidus is the core of the malformation, where high-flow, low-resistance shunting occurs directly between the arterial and venous systems. Depending on their location, AVMs can cause different complications, including hematuria, hypertension, high-output heart failure, and life-threatening hemorrhage [1]. 

The prevalence of AVMs depends on their location, with renal AVMs (rAVMs) having an estimated prevalence of approximately 0.04% [2]. rAVMs can be congenital or acquired. Congenital rAVMs are less common and occur due to abnormal embryonic vascular development during the fourth and 10th weeks of gestation [3]. Despite their embryonic origin, these lesions are usually diagnosed in adulthood [4]. Acquired rAVMs are more common and typically occur when trauma or iatrogenic injury creates an abnormal connection between the artery and vein [3]. These high-pressure shunts can cause venous aneurysms [1,5]. 

Several classification systems have been proposed for rrAVMs. More recently, the Yakes AVM classification system has been favored, as it is based on angiographic architecture and guides management strategy. Yakes Type I AVMs consist of direct artery-to-vein fistulae without an intervening nidus. Type II AVMs have multiple feeding arteries and a nidus, with draining veins that are nondilated (IIa) or dilated (IIb). Type III AVMs lack a true nidus and instead demonstrate multiple arterioles draining into a dilated venous sac, with one (IIIa) or multiple (IIIb) outflow veins. Type IV AVMs consist of diffuse, infiltrative arteriovenous channels forming a vascular network [6].

The clinical presentation of rAVMs varies, with hematuria being the most common symptom, often due to the rupture of small venules into the renal calyces as a result of increased intravascular pressure [3,7]. However, patients can also present with a bruit, hypertension, hemorrhage, or be asymptomatic/incidentally discovered during imaging for an unrelated reason.

rAVMs can be evaluated using several imaging techniques, each offering distinct advantages. Ultrasound (US), particularly with Doppler, often serves as the first-line modality, revealing a cluster of tortuous vessels with high-velocity arterial flow and early venous drainage [7]. Computed tomography angiography (CTA) provides excellent spatial resolution and can identify the nidus, feeding arteries, and early draining veins, especially helpful in acute settings [8]. Magnetic resonance angiography (MRA) similarly allows noninvasive visualization of the AVM’s vascular anatomy and can help differentiate vascular malformation from vascular soft-tissue tumors, although it may miss very small lesions [9]. Digital subtraction angiography (DSA) remains the gold standard, offering unmatched spatial and temporal resolution to precisely define the nidus, feeding and draining vessels, and hemodynamics, while also enabling endovascular treatment [3,8].

Different types of management are used for AVMs; the endovascular approach is gaining more traction as a less invasive alternative to surgical excision, having additional endovascular malformations creates a more challenging repair, such as a renal artery aneurysm [3,10,11]. One of the most frequently used techniques involves the use of coils, which primarily occlude the feeding vessels. However, because coils don’t address the nidus itself, this method could lead to failure of the procedure or recurrence of the lesion [3].

Here, we present a case of an rAVM with an associated venous aneurysm and the endovascular management using coil embolization and Onyx.

## Case presentation

A 53-year-old female patient with a history of hypertension, but no prior renal trauma, surgery, or biopsy, underwent a routine check-up with her primary care physician. She reported chronic, vague abdominal pain. On physical examination, an abdominal bruit was detected. Consequently, a renal duplex ultrasound with Doppler evaluation was ordered.

**Figure 1:**
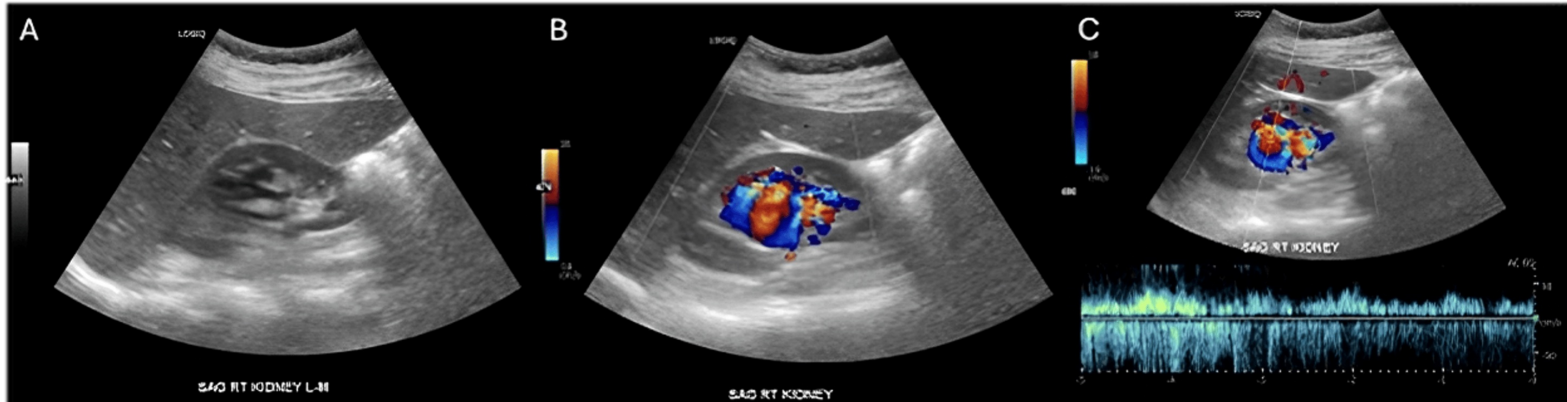
Duplex ultrasound of the right kidney. (A) Grayscale sagittal image showing an anechoic structure at the renal hilum. (B) Color Doppler image demonstrating internal turbulent flow. (C) Spectral Doppler waveform showing high-velocity, low-resistance arterial flow, initially thought to represent a renal artery aneurysm but later consistent with an arteriovenous malformation.

**Figure 2:**
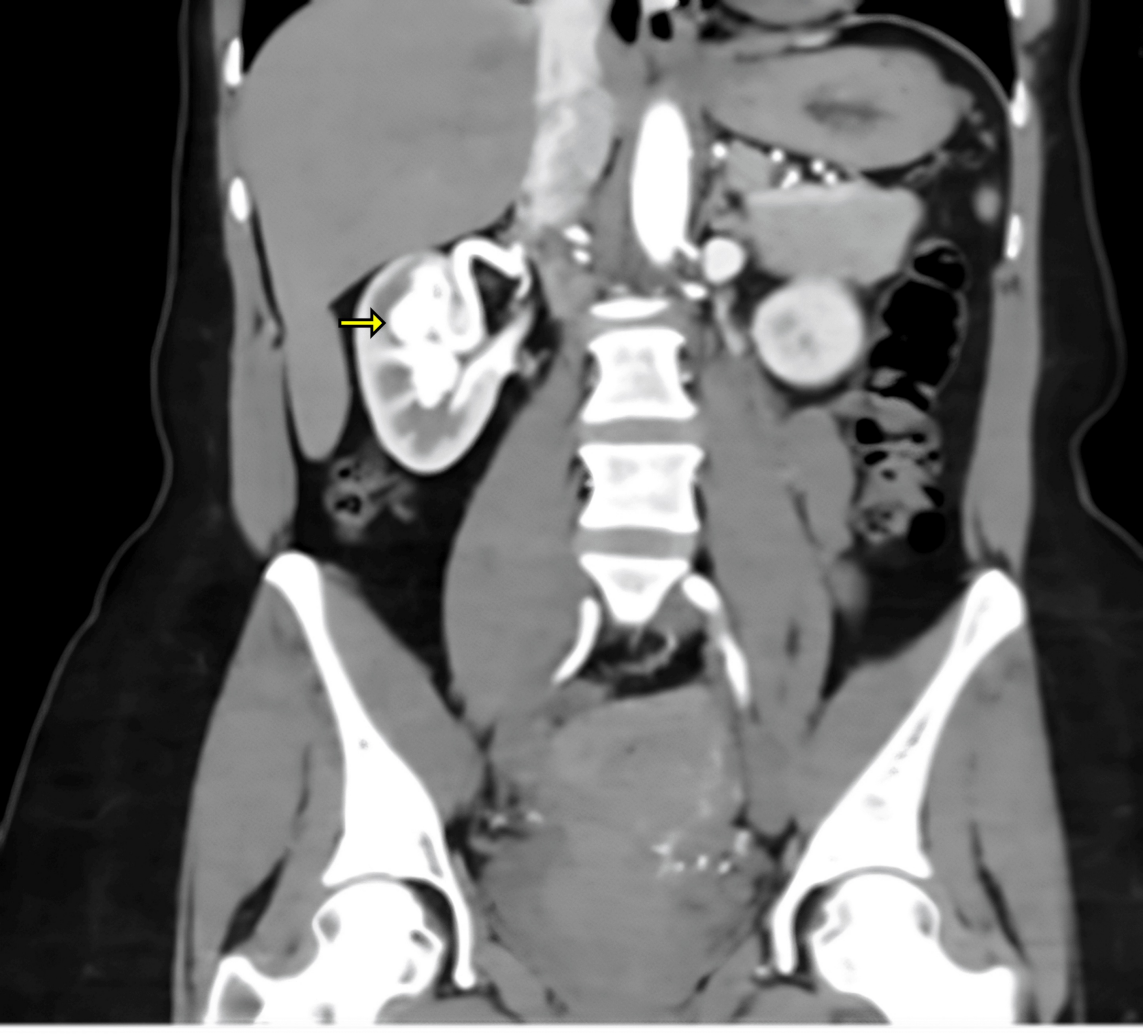
Coronal view of abdominal CT demonstrating the rAVM and venous aneurysm (yellow arrow) within the right renal hilum and bilateral double renal arteries. rAVM: Renal arteriovenous malformation

The ultrasound revealed a 4.3 cm ovoid, well-circumscribed, anechoic structure with brisk internal flow at the right renal hilum, thought to be a large renal artery aneurysm (Figure 1). To further characterize the observation, a CT angiogram was obtained, which revealed a duplicated right renal artery, with the upper pole artery markedly enlarged and connecting to the dilated and briskly enhancing upper pole vein compatible with a Yakes Type I rAVM with superimposed 3.8 cm venous aneurysm. Incidentally, the upper pole artery also exhibited a beaded appearance, suggestive of fibromuscular dysplasia, and an 8 mm saccular aneurysm (Figure 2).

The patient was initially evaluated by the vascular surgery team, who opted for nonoperative management. A six-month follow-up with a renal artery Doppler was planned.

At the six-month follow-up, repeat imaging was performed and raised concern for interval growth of the venous aneurysm, which showed a diameter of 4.4 cm, prompting multidisciplinary consultation. After discussion among vascular surgery, urology, and renal transplant services, the patient was referred to interventional radiology for endovascular management.

**Figure 3:**
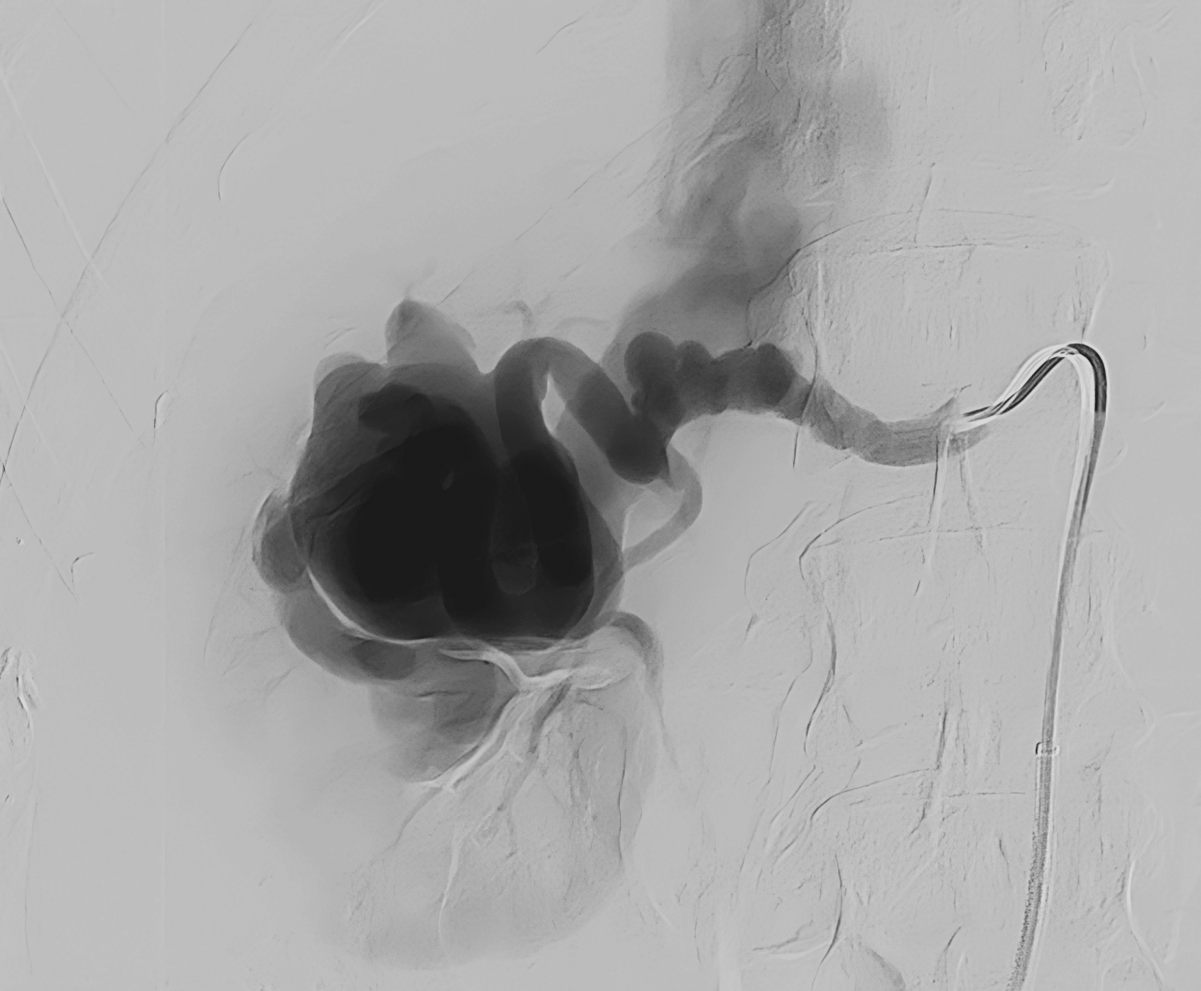
DSA of the right renal artery demonstrating a beaded arterial morphology and a large venous sac consistent with a high-flow arteriovenous malformation. DSA: Digital subtraction angiography

Right common femoral access was obtained in the conventional fashion with a micropuncture set (Cook Medical, Bloomington, IN, USA) with subsequent placement of a 6 French 45 cm long destination sheath (Terumo, Somerset, NJ, USA). Over a Bentson wire, a 5 French Shepherd’s hook catheter was used to select the superior right renal artery. DSA was performed (Figure 3), which redemonstrated the CTA findings detailed above.

After diagnostic angiography, the sheath was advanced over the wire into the ostium of the superior right renal artery. The 5 Fr catheter was exchanged for two microcatheters. First, a 2.0 Fr PX microcatheter (Penumbra, Inc., Alameda, CA, USA) was advanced over a 0.014” Chikai Black microwire (Asahi Intecc, Aichi, Japan) into the venous aneurysm. Second, a 6 mm × 20 mm Balt Eclipse (Balt Extrusion, Montmorency, France) dual-lumen balloon catheter was advanced over another 0.014” Chikai Black microwire (Asahi Intecc, Aichi, Japan).

The balloon occlusion catheter was intermittently inflated and deflated to control flow through the arteriovenous fistula to improve visualization of the angioarchitecture and reduce the risk of off target embolization. Via the distal microcatheter, the venous aneurysm was embolized with six Penumbra Ruby coils (40 mm and 36 mm) (Penumbra, Inc., Alameda, CA, USA). 

When intermittent angiography demonstrated significantly reduced flow within the sac, the PX microcatheter was removed. Then, with the balloon occlusion microcatheter inflated, the nidus was embolized with 0.7 cc of Onyx 34 (Medtronic, Irvine, CA, USA). 

**Figure 4:**
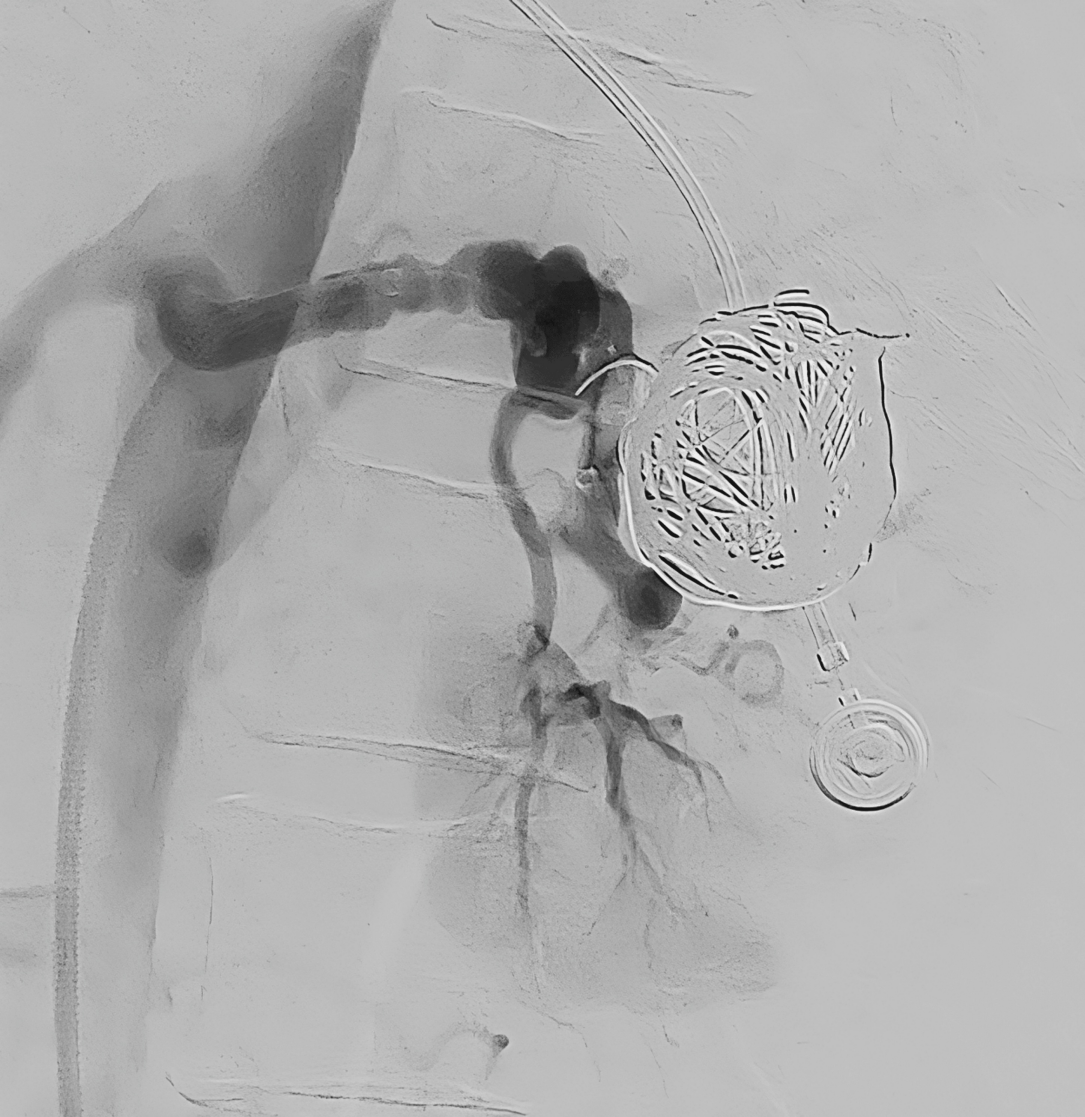
Post-embolization DSA demonstrating complete occlusion of the arteriovenous malformation and venous aneurysm. DSA: Digital subtraction angiography

Post-embolization angiography performed from the sheath demonstrated complete occlusion of the AVM and venous aneurysm without evidence of off-target embolization/renal infarction (Figure 4). 

The patient was monitored in the PACU for 24 hours without complications. She remained stable, denied pain, and exhibited no evidence of bleeding. A Foley catheter drained clear yellow urine for the first 12 hours before removal. Blood pressure remained stable, and the patient was afebrile.

**Figure 5:**
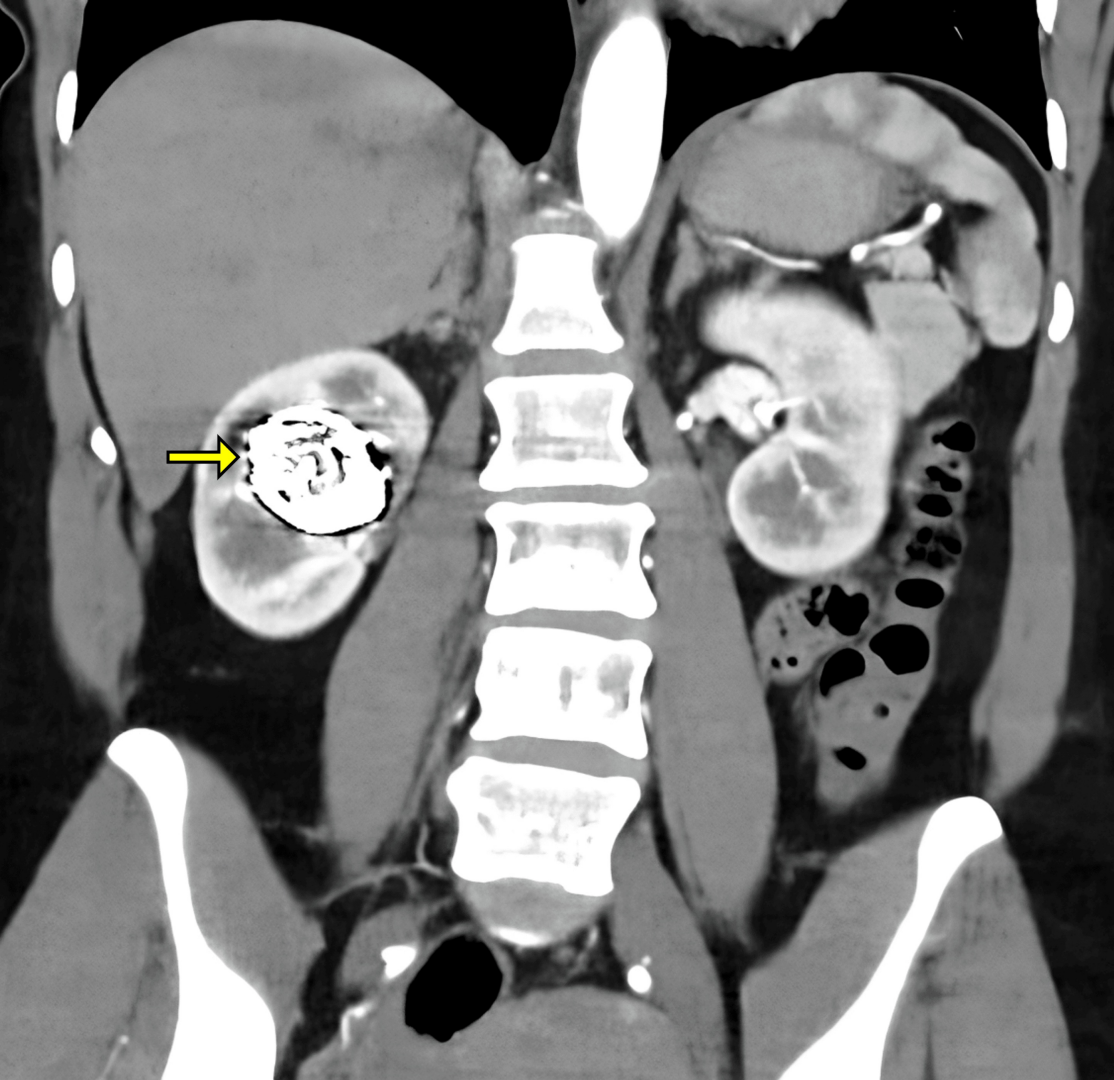
Postoperative abdominal CT follow-up showing well-preserved right renal perfusion and complete embolization of the aneurysm (yellow arrow).

A CTA performed two months after the embolization (Figure 5) demonstrated successful occlusion of the rAVM and venous aneurysm, with no residual arteriovenous shunting and well-preserved right renal perfusion without evidence of infarction. Notably, her hypertension resolved following the procedure, and she was able to discontinue her antihypertensive medications. Continued presence of fibromuscular dysplasia was noted in the codominant right renal arteries.

## Discussion

rAVMs are rare vascular anomalies, with an estimated prevalence of 0.04% in the general population [2]. In this instance, the rAVM was linked to fibromuscular dysplasia (FMD) in a duplicated right renal artery, a rare occurrence not often found in the literature [12]. While the clinical significance of this association remains uncertain, FMD may contribute to altered hemodynamics and vessel fragility, potentially playing a role in the development of vascular malformations such as rAVMs.

Management of AVMs has evolved significantly over the decades. Traditional options include surgical excision, partial/radical nephrectomy, auto-transplantation, and embolization of the affected segmental artery, approaches that carry high morbidity and risk of kidney infarction with loss of functional parenchyma [13,14]. In recent years, endovascular management has gained traction due to its lower morbidity, mortality, complication rates, and shorter hospital stays [3,15,16]. A recent retrospective study found that embolization of congenital renal AVMs significantly reduced complications compared to observation, with rupture occurring only in untreated Type I lesions, supporting early intervention even in asymptomatic patients [17]. In our patient’s case, surgical management would have required nephrectomy with ex vivo repair and reimplantation, a highly morbid approach. Instead, the endovascular intervention allowed for successful treatment with minimal risk and subsequent discharge within 24 hours of the procedure.

The main goal of endovascular management of rAVMs is to permanently close the shunt/nidus. Many embolic agents are available for this purpose, ranging from mechanical occlusive devices like coils and plugs to liquid embolics like ethanol, n-butyl cyanacrylate (NBCA) glue, and Onyx. Each embolic method has its pros and cons [3-5].

Coils, particularly detachable coils, and plugs are easy to visualize and can be placed with precision. However, they cannot fill multiple small vessels and may not penetrate the small vessels seen in type II and type III rAVMs. Additionally, in high-flow situations, they may fail to achieve complete occlusion [3,4].

Ethanol denatures proteins, resulting in irreversible endothelial damage and thrombosis. It can penetrate a nidus; however, it has to dwell within the vascular network long enough to induce damage, which can be challenging in high-flow states. Moreover, it is radiolucent and, thus, cannot be visualized, which increases the risk of incomplete or off-target embolization [10].

NBCA glue can also penetrate small niduses and mechanically occlude them. Theoretically, the concentration of the glue/ethiodized oil mixture can be used to alter the polymerization time and, thus, the level of occlusion. However, this precision is challenging to achieve in practice, particularly in high-flow lesions with a significant risk of embolic shunting. Moreover, there is a small but real risk of gluing the catheter into the feeding vessel [16].

Onyx, or 6% ethylene vinyl alcohol copolymer dissolved in dimethyl sulfoxide and suspended in micronized tantalum powder, has been used for the management of brain AVMs and, more recently, visceral AVMS [15,16,18-20]. It behaves like a viscous gel, allowing for increased control and lowered risk of off-target embolization. Moreover, it is cohesive, not adhesive; so, there is no risk of microcatheter entrapment. Its main drawbacks are cost and a learning curve for deployment.

We employed a multimodal embolization technique that was specifically developed for this intricate renal AVM in this instance. Balloon occlusion was strategically used to restrict inflow, improve visibility, and enable the placement of coils exactly where they needed to go in the venous outflow. These coils functioned as a scaffold, allowing for the controlled injection of Onyx to accomplish effective occlusion while preserving renal function.

Following the procedure, the patient's hypertension resolved, allowing for discontinuation of antihypertensive medication. In addition, no abdominal symptoms such as hematuria were observed postoperatively or during follow-up. The abdominal CTA performed during follow-up demonstrated a successful occlusion of the rAVM and the venous aneurysm with preserved right renal perfusion, without evidence of infarction. This highlights the clinical and technical success of the intervention.

## Conclusions

This case report demonstrates the feasibility and effectiveness of an endovascular approach for a complex renal artery aneurysm associated with an AVM. The patient's anatomical features, including bilateral renal artery duplication and concurrent fibromuscular dysplasia, increased procedural complexity. Additionally, by combining balloon-assisted coil embolization and Onyx injection, complete occlusion of the lesion was achieved while preserving normal renal perfusion and function, which should be monitored after the procedure and during follow-up with non-invasive imaging, assessment of blood pressure, and serum creatinine. These findings highlight the potential of minimally invasive endovascular strategies in treating complex renal vascular lesions. Further research, including larger case series with longer follow-up, is necessary to better define this approach's role in clinical practice.
